# On the Treatment of Phthisis by Iodine Inhalations

**Published:** 1854-05

**Authors:** M. Piorry


From the Virginia Medical and Surgical Journal.

*On the of Treatment of Phthisis by Iodine Inhalations.* By M. Piorry.

M. Piorry read an interesting paper on this subject to the French Academy of Medicine, (Jan. 24th, 1854,) from which we take the follwing extracts:

The majority of remedies hitherto employed in phthisis have been almost or entirely useless. Various medicinal agents have been vaunted as effiaccious in this disease, and then, after a few trials, experience has condemned them, and they have been abandoned and forgotten. Will the preparations of Iodine share the same fate? There is reason to hope that this will not be the case, and it is the design of this memoir to set forth the facts which authorize this hope.

I was induced to employ iodine and the vapours of iodine in the curative treatment of pulmonary phthsis by the following circumstances. It was known that iodide of potassium possessed a real and even prompt efficacy in chronic ostitis and periostitis, in scrofulous glandular enlargements, and in many other affections more or less allied with tuberculosis; M. Deyne, an interne of my service, and I, concluded that this remedy would be useful in phthsis (pneumophymie.) The results of our experiments were very satisfactory. A striking amelioration took place in many of our patients, and this amelioration was real, for of the patients mentioned in my work on Practical Medicine, three or four are still living in the enjoyment of good health.

After the successful treatment of hydrocele and tuberculous disease of the testicle by iodine injections, it was natural to attempt to obtain similar results in pulmonary excavations. It would have been difficult, if not impossible, at all events it would have been extremely rash to have injected tincture of iodine into the air-passages. We therefore bethought ourselves of the vapour of iodine. In hospital practice it was necessary to select the simplest methods of inhaling iodine. One or two scruples of iodine was accordingly placed in a wide-mouthed jar of the capacity of a quart; the vapour of it was disengaged spontaneously with more or less rapidity according to the degree of heat and moisture of the atmosphere.

When we used the tincture of iodine, we poured from one to three ounces in the jar, and heated it until the vapours of alcohol and iodine were liberated.

The patients breathed the air contained in these recipients, and charged with alcoholic and iodine vapour. One inspiration at a time is sufficient, but it should be deep, as when a sigh is heaved. Such an inspiration produces little irritation of the air-passages; it should be repeated one or two hundred times every day, at intervals, for several successive inspirations produce pain in the larynx and bronchi, and cough.

Even during sleep the patient should inhale iodine. For this purpose several saucers, each containing one scruple of iodine, should be placed about the pillow. At the hospital, we attach numerous phials of iodine to the iron frame which supports the bed curtains. The air thus becomes saturated with iodine; the starched curtains are colored blue, and the iron of the bedsteads assumes different tints under the action of the iodine.

If a moist starched paper is interposed between the jar containing iodine and the patient's mouth as he takes an inspiration, it turns blue; if the same air, after traversing the lungs is breathed upon the paper, it causes no change. The inference from this fact which I have observed very frequently, is that the iodine which entered the lungs is absorbed there, during the brief sojourn of the air in the air vesicles.

The majority of the patients subjected to this treatment at *La Pitie*, *La Charite*, and in my private practice, took also from twenty to sixty grains of iodide of potassium daily. In all those cases in which the extent of the lesions rendered it probable that adhesions, or that remarkable supplementary circulation so well described by Natalis Guillot, existed between the pulmonary and costal surfaces, we had recourse to frictions with tincture of iodine diluted with 19 parts of water. The patients were placed, in some cases, under other modes of treatment: 1. Under the use of tartar emetic in small doses, the fifth of a grain, for example. This heroic remedy was employed chiefly in those cases in which mucous, puriform, or purulent liquids accumulated in the bronchi, and produced a tendency to asphyxia or hypoxæmia. 2. Under the use of astringents, when the state of the intestinal canal required it; albumen, opiates, phosphate of lime, subnitrate of bismuth, etc., were employed with this object, but their use was discontinued as soon the diarrhœa (enterozhæa) was suppressed. 3. Under the use of quinine; in large doses when the spleen was congested; in small doses when there was simply a nightly exacerbation of fever depending upon the entrance of pus or softened tuberculous matter into the circulation. 4. Upon a nutritious and reparative diet; a very important point, for surely, if I was called upon to choose between hygienic precautions and the whole category of remedies besides iodine, I should give the preferenee to a good regimen. 5 Belladonna, opium, and other narcotics were employed, though rarely, to moderate the cough.

The cases which I have treated have not required the use of setons, issues, permanent blisters, or moxas, and I have not been able to comprehend the utility of these artificial pyogenic lesions in a disease in which the formation of pus is a disastrous accident.

Almost all of the patients remained in Paris. They were not sent to Nice or Pisa or other parts of Italy, a country where phthisical patients, coming from the north, in spite of all that has been said, recover no faster and no better than elsewhere.

Thirty-one patients have been subjected to the treatment thus described during the past two years. They all presented, in different degrees, the symptoms commonly attributed to pulmonary phthisis; that is, cough with puriform expectoration, hectic fever, emaciation; the majority of them suffered from diarrhœa, connected probably with tuberculous ulcerations; in many the larynx appeared to be involved in phymic disease; the majority had spit blood, (pneumoerhæmia.)

All of these subjects presented marked dullness at the summit of the lungs, either under the clavicle or at the superior scapular region. In most cases there was a hardness at these points, perceptible to the finger. Ordinarily it was possible to define the diseased structure accurately, and to distinguish the parts in which there was great condensation from those whieh had undergone less alterations of structure. In some cases a *bruit hydraerique* could be heard.*

In every case the stethoscopic signs were as positive as those revealed by plessimetry. At the points at which dullness and resistance had been noted, the ear recognized rude or tubal respiration, and more or less resonance of voice. In many cases large cavities were indicated by loud gurgling, cavernous respiration, and pectoriloquy. Each patient expectorated round, opaque, phy-

---

* The sound obtained by percussing over a cavity containing air and liquid Percussion over the cœcum during typhoid fever often gives excellent examples of it. —Trans.

oid sputa, the amount of which corresponded to the extent of the disease as determined by other methods of exploration.

I desired to appreciate the effects of iodine with precision, and therefore I did not trust to the indications of plessimetry. I ordered charts, on which were described exact delineations of the diseased parts, and representations of the variations in sound upon percussion which occurred from day to day. In casting the eye over these figures, it will be seen that after four, six or twenty days, six weeks, or three or four months of the iodine treatment, there was in almost every case a diminution in the extent of the surface over which there was at first feebleness of respiration, dullness, resistance, etc ; that, at the same time, the stethoscopic signs indicated an amelioration in the condition of the condensed portions of lung. This result did not occur only in those patients who were slightly diseased, but in almost every case. Numerous patients with cavities in the lungs were apparently cured. The ultimate results were as follows: Decided amelioration in the symptoms and anatomical characters in 20 patients. Disappearance of the anatomical characters and of most of the symptoms in 7 cases. Death, with or without amelioration, in 4 cases.

After some reflections upon the possible mechanism by which iodine operated in the cure of phthisis, M. Piorry concluded with the following propositions:

1. The inhalation of the vapour and tincture of iodine are useful in the cure of phthisis, [pneumophymie.]

2. In many cases such inhalation is followed by a diminution in the extent of the indurated parts surrounding tuberculous deposits, and an amelioration in the general symptoms;

3. It is probable that tubercle itself disappears under the influence of iodine inhalations;

4. That inhalations of the tincture of iodine may promote the cure of tuberculous cavities;

5. That after the softening of tubercles, the resulting cavities may cicatrize spontaneously;

6. That compression of the thorax over the points of disease indicated by percussion and auscultation, may contribute to the cure of the local lesion, and to the prevention of pyæmia;

7. That iodide of potassium administered internally, and frictions with diluted tincture of iodine over adherent portions of the lung, are also of great utility.

If, added M. Piorry, any useful therapeutical facts have been brought out in the preceding essay, I would observe that science and humanity are indebted for them to the progress of accurate diagnosis.

After this memoir was read, Dr. Londe observed that vapours of iodine was not the only remedy which could be inhaled with advantage in phthisis; he had employed arseniate of soda inhalations with great success. M. Caventu remarked that this salt was not volatilizable, and that if it had been used in fumigations, that arsenius acid vapours alone had been inhaled. M. Chatin observed that some physicians considered goitre and phthisis antagonistic, and that iodine was the real remedy for goitre. M. Bricheteau complained that M. Piorry had not alluded to his successful treatment of phthisis by tartar emetic.

M. Moreau regretted that Professor Piorry deemed it necessary to veil his ideas in language differing from ordinary scientific nomenclature; the terms *phymie*, *pneumophymie and phymopneumonie*, which M. Piorry employed instead of the terms, *tubercle*, *phthisis*, *t berculous pneumonia*, etc., produced a deplorable confusion in the m nd of the hearer. There was no reason, Prof. Moreau said, to encumber language with neologisms when there were already names for everything.

M. Piorry replied, with huge dignity, that an author who had published a work in eight volumes which had become a classic, had a right to use the nomenclature employed in that work. M. Moreau was not ignorant that Hippocrates employed the word *phymie;* it would be very unfortunate if in such an assembly, one could not be understood when employing words derived from the Greek.

